# Distribution, abundance, population structures, and potential impacts of the invasive snail, *Tarebia granifera* in aquatic ecosystems of north‐eastern South Africa

**DOI:** 10.1002/ece3.11544

**Published:** 2024-06-25

**Authors:** Ruan Gerber, Johannes J. Pearson, Victor Wepener, Wynand Malherbe, Lizaan de Necker

**Affiliations:** ^1^ Water Research Group, Unit for Environmental Sciences and Management North‐West University Potchefstroom South Africa; ^2^ South African Institute for Aquatic Biodiversity (NRF‐SAIAB) Makhanda South Africa; ^3^ Animal Ecology, Global Change and Sustainable Development, Department of Biology University of Leuven Leuven Belgium

**Keywords:** biodiversity, invasive species, Mollusca, population densities, quilted melania

## Abstract

Aquatic ecosystems globally have been invaded by molluscs. *Tarebia granifera* is a highly successful invader, often becoming the dominant aquatic invertebrate species in an invaded ecosystem. Resultingly, it has been suggested that *T. granifera* may have severe negative impacts on these invaded ecosystems. Limited information is available regarding the population structures and densities of *T. granifera*, particularly in invaded countries such as South Africa, and information on this could assist in developing management and control strategies for this invasive species. The present study aimed to assess the current distribution, densities, and population structures of *T. granifera* in invaded habitats on the Limpopo and Phongolo River systems in South Africa. This was accomplished by collecting aquatic molluscs from sites across these systems. Water quality parameters were measured at each site and water samples were collected for chemical nutrient analyses. The density of snails was determined for each site and the population size and structure as well as birth rate was calculated for *T. granifera. Tarebia granifera* was found to be the dominant molluscan species in habitats where it was present and all size classes from newborn to mature adults were found throughout at some of the highest densities globally. Worryingly, native molluscan species, were often absent or in much lower densities than reported in literature at sites where *T. granifera* was present, suggesting a negative effect on the native molluscan density and diversity. Contrary to most previous studies, there were no significant correlations between *T. granifera* and the selected water quality parameters. Higher densities and newborn recruitment of *T. granifera* were observed in the spring than in autumn, likely in response to shifts in environmental conditions. This study provides crucial insights into the population structure, densities, and impacts of *T. granifera* in invaded habitats, particularly for relatively newly invaded regions such as southern Africa.

## Graphical abstract

The study investigates *Tarebia granifera*, a highly successful aquatic invader, in South Africa's Limpopo and Phongolo River systems. The study revealed that *T. granifera* dominates in habitats where it is present, negatively impacting native molluscan species. Interestingly, the study did not find significant correlations between *T. granifera* and water quality parameters and observed higher densities during spring, providing valuable insights for managing this invasive species in newly invaded regions like southern Africa.

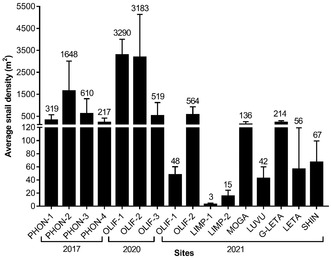

## 1 INTRODUCTION

*Tarebia granifera* (Lamarck 1822) also known as the quilted melania, is a prosobranch (gill‐breathing) gastropod native to Southeast Asia that has successfully invaded predominantly tropical and subtropical aquatic ecosystems across several continents (see review by Pearson et al., In press). Numerous water bodies in South Africa have been invaded since it was first reported in the country (Pearson et al., In press). These include the aquatic ecosystems of the tropical and subtropical regions of KwaZulu‐Natal, Limpopo, and Mpumalanga in South Africa (Dube et al., 2017; Malherbe, 2018).

*Tarebia granifera* is a highly resilient and opportunistic species as it can migrate upstream in fast‐flowing water (Appleton et al., 2009), tolerate a wide salinity and temperature range, and has no known natural predators in South Africa (Miranda & Perissinotto, 2012). Furthermore, this snail can flourish in highly disturbed environments (Jones et al., 2017). These adaptations enable it to rapidly disperse and invade a wide variety of aquatic habitats in a relatively short time (Appleton et al., 2009; Jones et al., 2017). The rate and range of *T. granifera* invasion is a matter of great concern and needs to be investigated and closely monitored (Appleton et al., 2009; Pearson et al., In press; Weyl et al., 2020). As *T. granifera* is generally a dominant invertebrate species in invaded aquatic ecosystems (Appleton et al., 2009; Pearson et al., In press; Weyl et al., 2020), there is a high probability of environmental harm, an innate threat due to their ability to reproduce rapidly (Kesner & Kumschick, 2018). This parthenogenetic species can give birth to one fully developed juvenile snail every 12 h (Appleton et al., 2009).

Research work on the impacts of this invader on native snail community structures and biodiversity is therefore urgently needed (Weyl et al., 2020). Studies on the population dynamics of an invasive species can provide information on how these species interact with newly invaded ecosystems (Miranda et al., 2011b). Over time, fluctuations in the population densities and size structures of *T. granifera* can reveal how this invasive snail can survive and recover during unfavourable conditions, such as drought, flooding, or increased water salinity (Miranda et al., 2011b). Previous studies from South Africa reported differences in *T. granifera* density due to differences in water quality (Jones et al., 2017; Makherana et al., 2022). However, little is reported on their effect on invaded ecosystems in the country.

Recent studies regarding snails recommend reporting on snail densities and population size structures to understand the effects of the snail species on ecosystem function and community structures of freshwater ecosystems (Ruehl & Trexler, 2011; White et al., 2007). The density of a species can affect the biology of numerous other species in the same ecosystem (Conner et al., 2008). For example, a decrease in the growth rate of native species can delay sexual maturity (reduced reproduction), causing an increase in mortality, and the displacement or total eradication of native snail species (Appleton et al., 2009; Jones et al., 2017; Miranda et al., 2011b; Pearson et al., In press). Many of the ecosystem changes caused by invasive species are irreversible due to the difficulty of removing such invaders from invaded habitats (Kesner & Kumschick, 2018). The ecological drivers behind the successful spread of *T. granifera* have not been established thus far and shifts in the density and population size patterns of this invader, as well as its management status, are understudied (Makherana et al., 2022). A better understanding of the population dynamics of this invader can help predict how they will react towards environmental changes, which can lead to the development of management and control strategies (Makherana et al., 2022; Miranda et al., 2011b).

The overarching aim of this study was to investigate the current distribution, densities, and population size structures of *T. granifera* at selected sites in rivers in north‐eastern South Africa and the potential environmental drivers thereof as well as their potential impacts on native aquatic mollusc diversity in the region to answer the following questions: (1) Are *T. granifera* densities and population size structures evenly distributed across sites and river systems in north‐eastern South Africa?; (2) Are *T. granifera* densities and population size structures driven by environmental variables, that is, water quality?; (3) Is benthic mollusc diversity and distribution negatively impacted by the presence, density, or population size structures of *T. granifera* in the study region?

The hypotheses around the aforementioned questions are as follows: (1) *T. granifera* densities and population size structures will reflect environmental conditions, with higher densities and more varied population size structures observed at sites and systems with more favourable environmental conditions; (2) the densities and population size structures of *T. granifera* will be significantly influenced by environmental variables, with higher densities and less varied population size structures in areas with higher nutrients, pH, and conductivity; (3) The presence, density, and population size structures of *T. granifera* will have a negative impact on benthic mollusc diversity and distribution in the study region. Areas with higher densities and a larger fraction of mature, adult *T. granifera* will exhibit reduced benthic mollusc diversity and altered distribution patterns compared with areas with lower densities or without *T. granifera* presence.

## 2 MATERIALS AND METHODS

### 2.1 Study region

**FIGURE 1:**
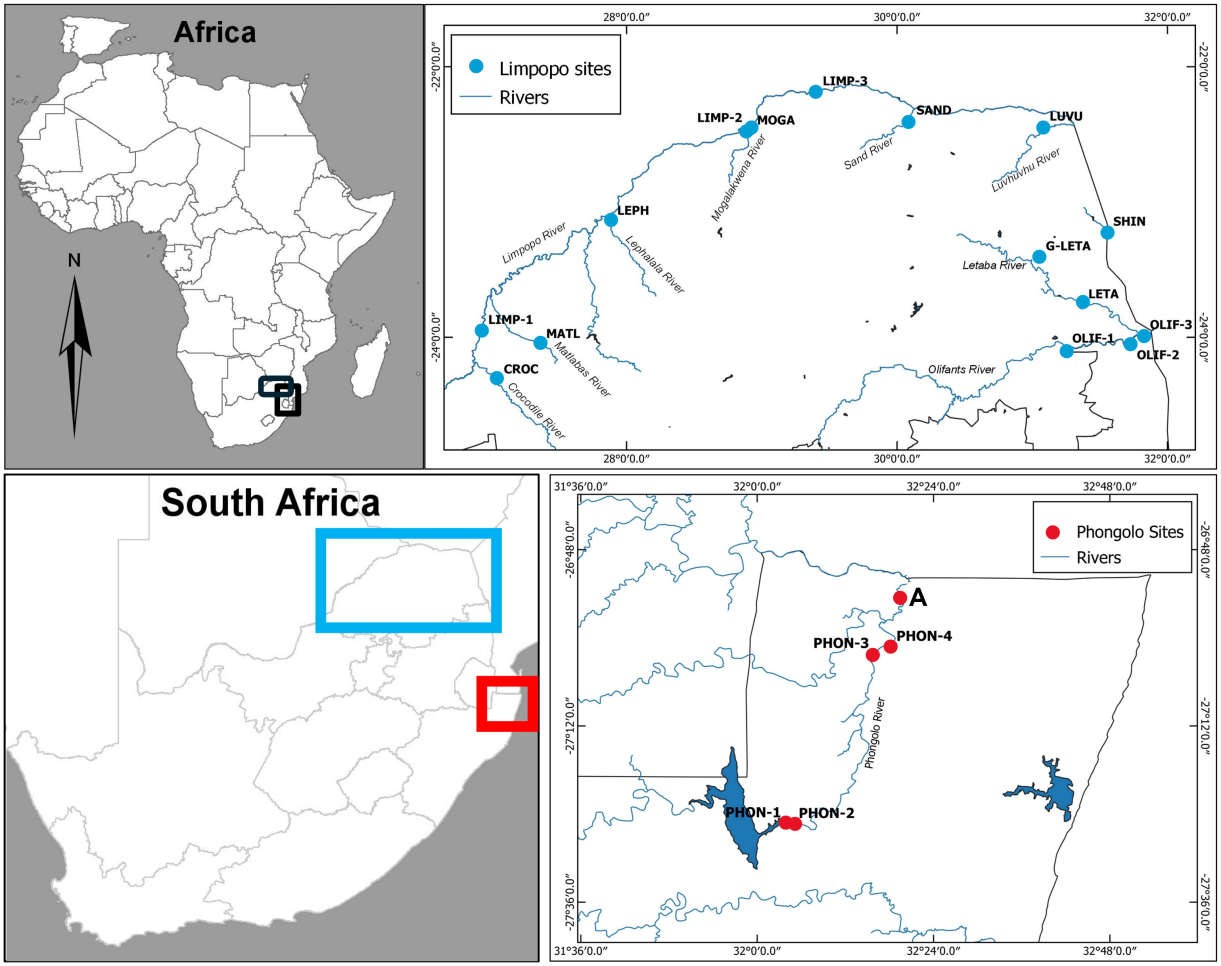
Map indicating all selected sites on the Phongolo River system (red points) and the Limpopo River system (blue points).

Nineteen sites were selected for sampling in this study based on known distributions of aquatic snails (invasive and native) and accessibility to sites (Figure 1). Four sites were sampled on the lower Phongolo River in northern KwaZulu‐Natal (August–September 2017), South Africa, and a total of 15 sites were sampled on the Limpopo River and its tributaries (Limpopo River system), in the Mpumalanga and Limpopo provinces of South Africa (April–May 2021). Two sites in the Limpopo River system on the Olifants River (OLIF‐1, OLIF‐2) were sampled in consecutive years (2020 and 2021). Site coordinates and other relevant site‐related information are provided in Table 1.

**TABLE 1:** Site names, sampling dates, location, coordinates and elevation data for sampling sites on the lower Phongolo and Limpopo River systems sampled during the current study.

Site code	Sampling date	River	Coordinates	Elevation (m.a.s.l.)
PHON‐1	31/08/2017	Phongolo	−27.421003, 32.067122	~140
PHON‐2	31/08/2017	Phongolo	−27.42268, 32.08153	~73
PHON‐3	31/08/2017	Phongolo	−27.03677, 32.26647	~33
PHON‐4	02/09/2017	Phongolo	−27.02063, 32.30313	~32
CROC	21/04/2021	Crocodile	−24.31417, 27.04614	885
LIMP‐1	22/04/2021	Limpopo	−23.9485, 26.93123	857
MATL	23/04/2021	Matlabas	−24.05186, 27.35964	916
LEPH	24/04/2021	Lephalala	−23.14128, 27.88503	794
LIMP‐2	25/04/2021	Limpopo	−22.45519, 28.90175	631
MOGA	26/04/2021	Mogalakwena	−22.47344, 28.91950	636
LIMP‐3	27/04/2021	Limpopo	−22.18383, 29.40519	511
SAND	28/04/2021	Sand	−22.39928, 30.09942	447
LUVU	29/04/2021	Luvuvhu	−22.44444, 31.08344	249
OLIF‐1	30/09/2020 05/05/2021	Olifants	−24.08642, 31.25094	278
OLIF‐2	04/10/2020 03/05/2021	Olifants	−24.05214, 31.72878	185
OLIF‐3	03/10/2020	Olifants	−23.99071, 31.82659	130
G‐LETA	06/05/2021	Great Letaba	−23.677528, 31.098639	318
LETA	04/05/2021	Letaba	−23.943389, 31.735111	264
SHIN	01/05/2021	Shingwedzi	−23.22194, 31.55492	241

The Phongolo River was selected as it is known to be invaded by *T. granifera* (Dube et al., 2017, 2019; Smit et al., 2016). Sites were selected within the main channel of the Phongolo River and its floodplain, stretching downstream from the Pongolapoort Dam to before the confluence with the Usuthu River (refer to Figure 1). Site selection was based on study sites used as part of previous studies in the region (Dube et al., 2017; de Necker, 2019; de Necker, Gerber et al., 2022). The Phongolo River system is the largest floodplain system in South Africa (Acosta et al., 2020) and is often the most southern distribution locality for many lowveld tropical freshwater species, making it an ecologically significant area. The Limpopo River is one of the largest rivers (~1750 km) in southern Africa, its basin draining an area of ~415,000 km^2^ across South Africa, Botswana, Zimbabwe, and Mozambique (Mosase & Ahiablame, 2018). The Olifants River is one of the most polluted and threatened river systems in South Africa due to the deteriorating water quality caused by mining, industrial, and agricultural activities (Gerber et al., 2015). Furthermore, the Olifants River was selected as it is known to be invaded by *T. granifera* (Verhaert et al., 2017).

### 2.2 Mollusc sampling and identification

Each of the 19 selected sites was sampled for aquatic benthic molluscs (including snails and bivalves/clams), using a square metal‐frame benthic sampler (30 × 30 cm, 2 mm mesh size). The sampler was used to scoop up sediment and sieve through the benthic zone of the river. Random samples, each comprising three replicates consisting of 10 scoops per replicate (30 sampler scoops) covering a total area of 2.7 m^2^ were collected. Sampling occurred randomly within the site areas, avoiding deep waters due to the presence of crocodiles (*Crocodylus niloticus*). Samples were preserved with 70% ethanol in plastic jars and transported back to the laboratory and molluscs were identified to species level according to Appleton (2002) and Fry (2021). Only individuals that were considered “alive” (i.e., shell closed by operculum) at the time of sampling were counted and the density of each species per site (number of individuals sampled/m^2^) was determined. This sampling was followed in order to provide the relevant data to answer the three core questions of this study.

### 2.3 *Tarebia granifera* population size structures

To determine the population size structures for *T. granifera*, samples were processed through sieves and snails divided into size classes according to shell length (Table 2). For this study, size classes were used to describe age groups based on the size of sexual maturity in the populations obtained from published literature. Samples were separated into size classes using a King Test (VB 200 300) sieve shaker with Clear Edge Test sieves. *Tarebia granifera* were divided into mature adults (>10.39 mm) that are considered able to reproduce, adults (5.28–10.39 mm) becoming sexually mature and starting to reproduce, juveniles (1.92–5.27 mm) not able to reproduce, and newborn snails (<1.9 mm) that were recently born (Appleton et al., 2009; Miranda et al., 2011b). The size class distribution of *Tarebia granifera* was assessed in order to provide the relevant data to answer the three core questions of this study.

**TABLE 2:** Size class distribution of *Tarebia granifera* in width and length measurements (mm) *n* = 1070.

Snail age group	Mature adults	Adults	Juveniles	Newborns
Shaker sieve size (μm)	4000	2000	1000	500
Snail width range (mm)	>4	4–2	2–0.5	<0.5
Minimum snail length (mm)	10.40	5.28	1.92	<1.9
Maximum snail length (mm)	>10.40	10.39	5.27	1.9
Snail abundance %	5.8	54.5	36.5	3.2
Snail length size class (mm)	>10.39	10.39–5.28	5.27–1.92	<1.92

#### 2.3.1 *Tarebia granifera* birth rate

Although not an original aim of this study or one of the core questions, the sampling regime did allow for the calculation of the birth rate for *T. granifera* from mature adult *T. granifera* snails (>10.4 mm) collected from the three sites sampled on the Olifants River in 2020. These snails gave birth for three consecutive days whilst housed in 1‐litre jars filled with filtered river water (Tolley‐Jordan & Owen, 2008) and fed with shredded lettuce (Schuster et al., 2014). To ensure snails had adequate oxygen, filtered river water from the site was changed once a day and snails were kept at room temperature away from direct light to reduce stress and overheating (Schols, 2019). The newborn snails were collected, and the birth rate was calculated:

Birth rate=Nb/Na/Nd.

Where Nb is the number of juvenile snails born, Na is the number of adult snails housed and Nd is the number of days the snails were housed.

### 2.4 Water quality analysis

Physicochemical water quality variables: pH, temperature (°C), electrical conductivity (EC) (μS/cm), total dissolved solids (TDS) (mg/L), dissolved oxygen (mg/L), and percentage oxygen saturation (%) were measured in situ at each site using Extech EC500 pH/electrical conductivity and Extech DO600 dissolved oxygen water quality meters. Water samples (250 mL) from each site were collected in triplicate for chemical nutrient analyses. Samples were preserved by freezing (−4°C) them on‐site using a portable camping fridge/freezer until analysis. In the laboratory, water samples were left to defrost at room temperature and analysed for the following chemical nutrient variables: Turbidity (FAU), SO_4_^2−^ (14791), NH_4_^+^ (14752), NO_3_^−^ (14773), NO_2_^−^ (14776), PO_4_^3−^ (14848), and Cl^−^ (14897) (mg/L) using the appropriate test kits (test kit catalogue numbers in parentheses) and standard protocols with a Merck Spectroquant Pharo 300 Spectrophotometer (de Necker et al., 2022). Selected water quality variables were assessed to evaluate whether benthic mollusc populations were significantly related to any of the measured variables.

### 2.5 Permits and ethical clearance

All samples were collected and processed with the necessary permits from the Limpopo Department of Economic Development, Environment and Tourism (Permit No. ZA/LP/107276), Ezemvelo KZN Wildlife (Permit No. OP 941/2016, OP 1075/2017 and OP 864/2021) and ethical clearance from the North‐West University AnimCare Animal Research Ethics Committee (Ethics No. NWU‐01539‐20‐A9).

### 2.6 Data analysis

#### 2.6.1 Evaluation of *T. granifera* densities and population size structures across sites

To assess whether *T. granifera* densities differed and whether population size structures were evenly distributed across the sampled sites and river systems a Chi‐square goodness of fit test and a Chi‐square contingency table were applied using relevant functions in Microsoft Excel, respectively.

#### 2.6.2 Evaluation of *T. granifera* densities and population size structures in relation to water quality variables

The relationship between *T. granifera* densities with water quality parameters was evaluated firstly using Pearson's correlation analyses in GraphPad Prism version 7.0.0., secondly for both density and each population size class, multiple regression analyses were performed separately to establish which of the measured water quality variables, if any, best predicts these *T. granifera* population parameters from the various sampled aquatic systems. For these specific multiple regression analyses, due to collinearity, both chlorides and sulphates were removed from the analysis. Analyses were done in SPSS version 18 (PASW Statistics, IBM).

#### 2.6.3 Evaluation of *T. granifera* densities and population size structures on benthic mollusc diversity

Potential significant differences in snail density for each of the species in each of the sampled systems were tested using a Kruskal–Wallis test for non‐parametric data and Dunn's post hoc test to determine specific significant (*p* < .05) pairwise differences between the densities of *T. granifera* versus the other snail species sampled from each system (de Necker, 2019; Stoline, 1981). Thereafter, multiple regression analyses were conducted (1) to rule out whether water quality variables predicted the densities of other benthic molluscs sampled concurrently with *T. granifera* at selected sites. For these specific multiple regression analyses, due to collinearity, both chlorides and sulphates were removed from the analysis. The second multiple regression analyses that followed were performed separately to establish which of the measured *T. granifera* population parameters, if any, best predicts population densities of other benthic molluscs sampled concurrently with *T. granifera* at selected sites. For these specific multiple regression analyses no collinearity was present between the variables. All analyses were done in SPSS version 18 (PASW Statistics, IBM).

## 3 RESULTS

### 3.1 *Tarebia granifera* presence

Aquatic molluscs were found to be present at 14 of the 19 sites and absent from five (CROC, MATL, LEPH, SAND, and LIMP‐3). Four species, two invasives: *T. granifera* and *Physella acuta* (Draparnaud, 1805) and two natives: *Melanoides tuberculata* (Müller, 1774) and *Corbicula africana* (Krauss, 1848) and a total of 29,378 individuals were sampled, counted and measured. *Tarebia granifera* was the only species that occurred at all 14 of these sites and also the dominant species, making up 26,178 individuals, followed by *C. africana* (1596 individuals), *M. tuberculata* (1456 individuals) and *P. acuta* (148 individuals).

### 3.2 *Tarebia granifera* density

**FIGURE 2:**
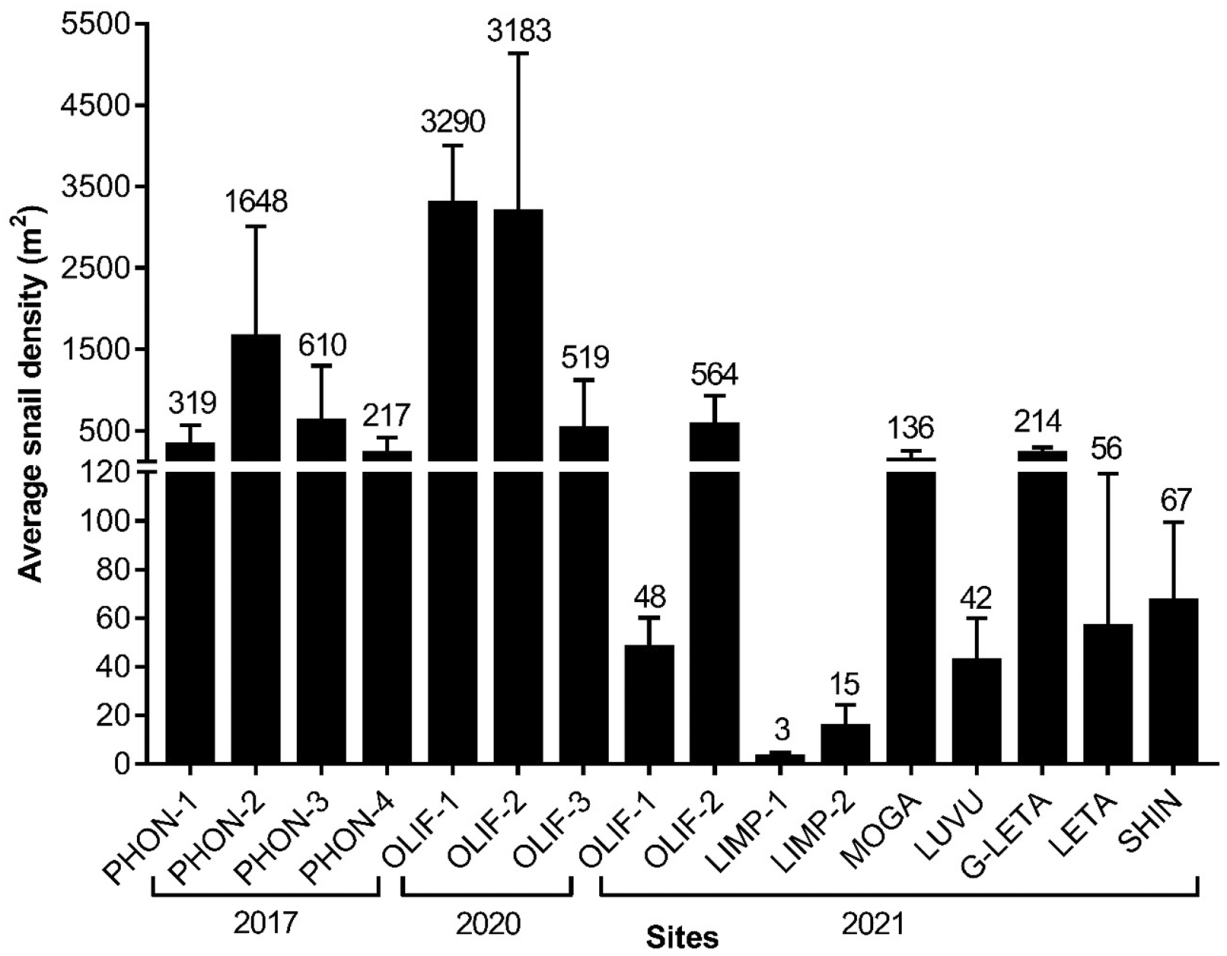
Average *Tarebia granifera* population density (±SD) sampled from the selected sites on the Phongolo River and Limpopo River system from 2017 to 2021.

The *T. granifera* densities at all sampled sites are illustrated in Figure 2. *Tarebia granifera* was present at all four sites sampled on the Phongolo River, with an average density of 699 individuals/m^2^. The PHON‐2 and PHON‐3 sites had the highest densities of *T. granifera* whilst the PHON‐1 and PHON‐4 sites had the lowest densities. The highest snail densities recorded during the entire study were of *T. granifera* from the OLIF‐1 and OLIF‐2 sites during the 2020 survey on the Olifants River. These two sites (OLIF‐1 and OLIF‐2) also had much lower densities of *T. granifera* in 2021. The three sites sampled on the Limpopo River had low densities of the invasive *T. granifera*. The highest density of the snail in this system was recorded at the second site on the Limpopo River (LIMP‐2) followed by the first site (LIMP‐1), whilst the LIMP‐3 site did not have any snails present. The sites on the Mogalakwena (MOGA) and Great Letaba (G‐LETA) rivers had higher densities of *T. granifera*, whilst the sites on the Shingwedzi River (SHIN), Letaba River (LETA) and the Luvuvhu River (LUVU) had lower densities of the invasive snail. The Chi‐square goodness of fit analysis revealed a random association of *T. granifera* densities across the sampled sites; *χ* ^2^(df = 15, *n* = 16) = 25,450, *p* < .0001. However, when the analysis was repeated at the system level (i.e., Phongolo River vs. Olifants River vs. Limpopo River) the Chi‐square contingency table revealed that *T. granifera* densities were associated with the system they were sampled from; *χ* ^2^(df = 9, *n* = 16) = 15.37, .1 > *p* > .05.

### 3.3 *Tarebia granifera* population characteristics

**FIGURE 3:**
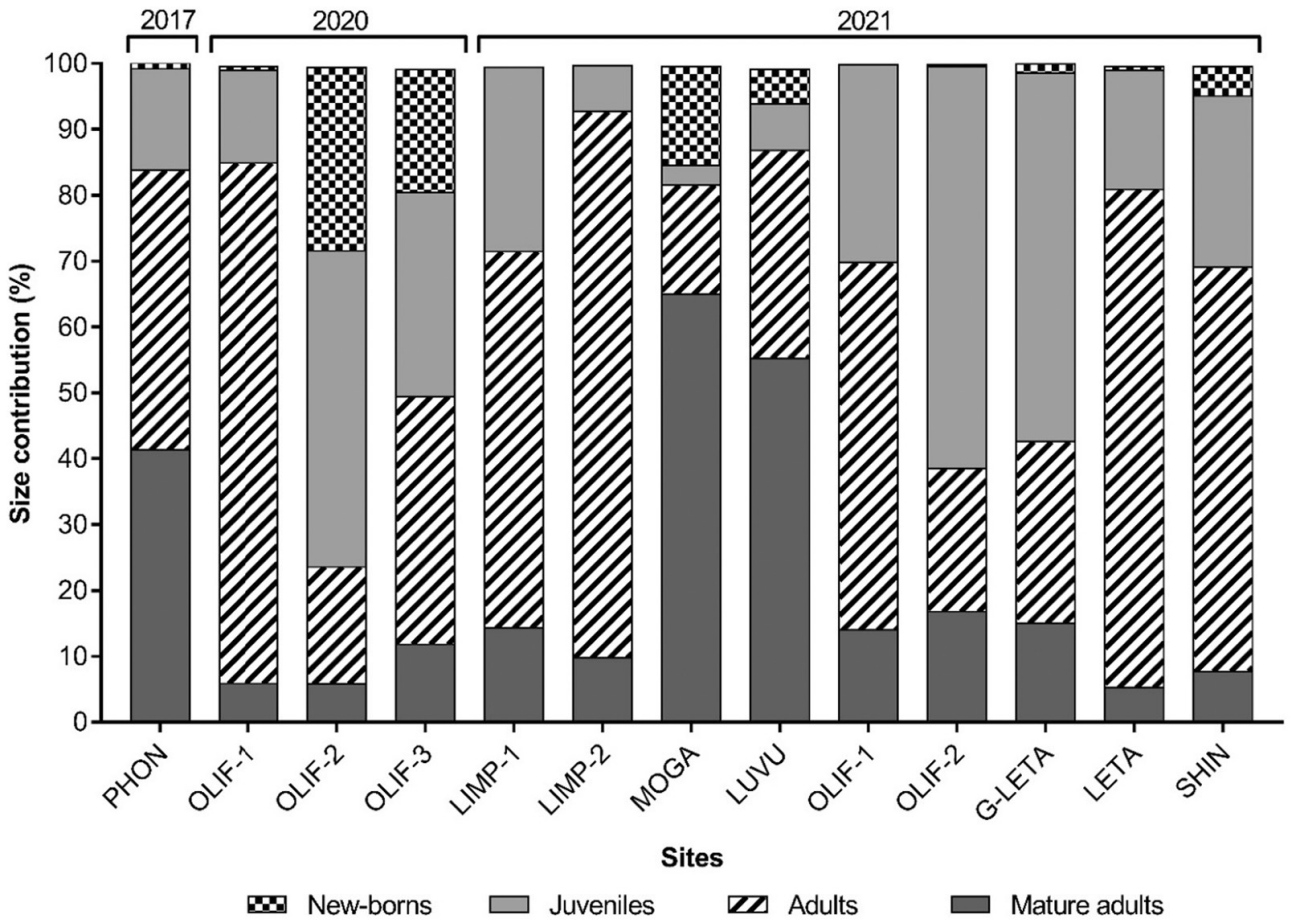
Percentage size contributions of *Tarebia granifera* populations from all selected sites sampled from 2017 to 2021. Snails were divided into mature adults (>10.39 mm), adults (5.28–10.39 mm), juveniles (1.92–5.27 mm), and newborns (<1.9 mm).

The average birth rate of *T. granifera* was 1.89 newborn snails per mature adult per day. *Tarebia granifera* population size structures from all sites sampled are illustrated in Figure 3. The *T. granifera* population from the Phongolo River (PHON) mostly consisted of adults and mature adults (~84%), whilst juveniles and newborns made up <16% of the population, respectively. The *T. granifera* population structures in the lower Olifants River were highly variable, for example, during the 2020 survey adults dominated at OLIF‐1 (79%), whilst juveniles and newborns were more abundant (~76%, respectively) at OLIF‐2. There was an even population distribution at OLIF‐3. The *T. granifera* population structures at OLIF‐1 and OLIF‐2 during the 2021 survey did not change in terms of size classes with adults and juveniles dominant at these sites. Notably, in 2021, no newborns were recorded at OLIF‐1 and very few at OLIF‐2.

On the Limpopo River, no newborn *T. granifera* were sampled and adult snails formed the largest part of the populations at both LIMP‐1 and LIMP‐2. The *T. granifera* population structure in the Mogalakwena River (MOGA) was dominated by mature adults and adults (~86%) and juveniles made up the smallest proportion of the population. The population structure from the Luvuvhu River site (LUVU) was also dominated by mature adults and adults (~87%), whilst juveniles and newborns had lower abundances. On the Great Letaba River (G‐LETA), populations mainly consisted of juveniles (56%) followed by adults and mature adults (~42%) with very few newborns. The *T. granifera* population structures of the Letaba – (LETA) and Shingwedzi River (SHIN) followed the same trend. Adults dominated (>60%), followed by juveniles, mature adults, and newborns, respectively.

The Chi‐square goodness of fit analysis revealed a random association of *T. granifera* size class distribution across the sampled sites; *χ* ^2^(df = 36, *n* = 13) = 1112.1, *p* < .0001. This was also the case when the analysis was repeated at the system level (i.e., Phongolo River vs. Olifants River vs. Limpopo River) the Chi‐square contingency table revealed that *T. granifera* size class distributions were randomly associated with the system they were sampled from; *χ* ^2^(df = 9, *n* = 4) = 180.1, *p* < .0001.

### 3.4 *Tarebia granifera* population variables in relation to water quality parameters

There were no significant correlations between *T. granifera* density and any of the water quality variables (Table 3; *p* > .05; *R* ^2^ < .06). Likewise, none of the multiple regression analyses run to predict *T. granifera* densities or size class contributions from the various water quality variables were able to significantly predict these population variables across sites: *T. granifera* densities – *F* (5, 10) = .205, *p* = .953, *R* ^2^ = .093; Adult *T. granifera* – *F* (5, 7) = .449, *p* = .803, *R* ^2^ = .243; Mature *T. granifera* – *F* (5, 7) = .237, *p* = .934, *R* ^2^ = .145; Juvenile *T. granifera* – *F* (5, 7) = .314, *p* = .890, *R* ^2^ = .183; Newborn *T. granifera* – *F* (5, 7) = 1.663, *p* = .261, *R* ^2^ = .543.

**TABLE 3:** Pearson correlations between *Tarebia granifera* density and water quality variables of sites sampled on the Phongolo River and Limpopo River system.

	Water quality variables							
	Temperature (°C)	Conductivity (μS/cm)	pH	Cl^−^ (mg/L)	SO_4_ (mg/L)	NO_3_ (mg/L)	NH_4_ (mg/L)	PO_4_ (mg/L)
*T. granifera* density (m^2^)	*r* = .034 *p* = .50	*r* = .021 *p* = .59	*r* = .0001 *p* = .95	*r* = .026 *p* = .55	*r* = .056 *p* = .38	*r* = .046 *p* = .42	*r* = .0054 *p* = .79	*r* = 0.009 *p* = .73

### 3.5 Molluscan communities

**FIGURE 4:**
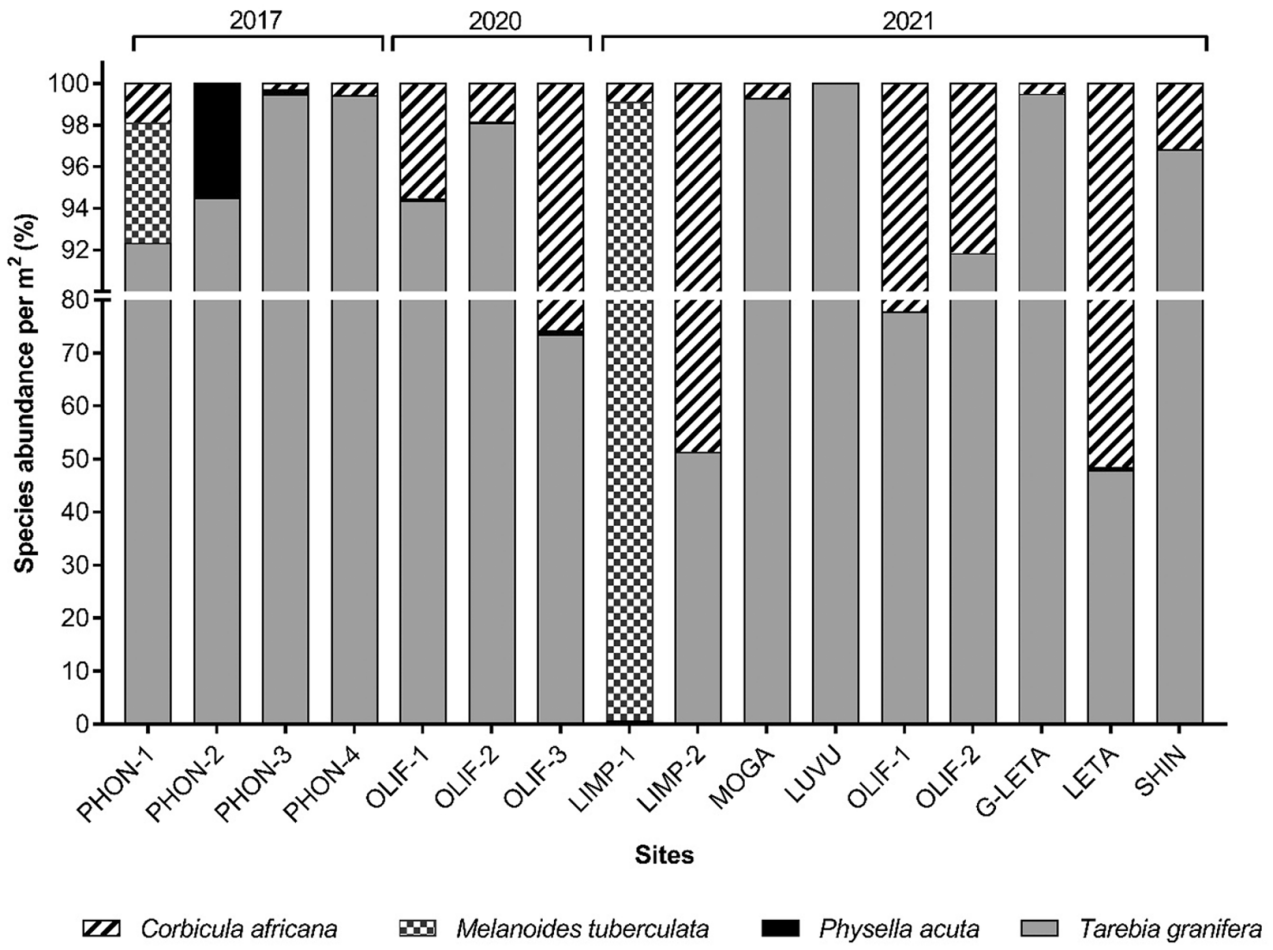
Percentage contribution of mollusc communities per site of all species sampled from the selected sites between 2017 and 2021.

The percentage contribution of mollusc species sampled at each site is illustrated in Figure 4. A total of four mollusc species (*T. granifera, C. africana*, *M. tuberculata* and *P. acuta*) were sampled from the Phongolo River. *Tarebia granifera* was the dominant species sampled on the Phongolo River and contributed to over 92% of the mollusc communities sampled in the river. The native *C. africana* was absent from PHON‐2 and made up less than 2% of the mollusc community at the other three sites and low densities (<eight individuals/m^2^). The invasive *P. acuta* was only present at PHON‐2 and PHON‐3, respectively. The highest density for *P. acuta* was recorded at PHON‐2 (96 individuals/m^2^), whilst only one individual/m^2^ was recorded at PHON‐3. The native *M. tuberculata* were collected only at PHON‐1, contributing to <6% of the mollusc community with a density of 20 individuals/m^2^ at the site.

From the three sites sampled on the Olifants River during the 2020 survey, three species (*C. africana*, *P. acuta* and *T. granifera*) were collected with a total average density of 2480 individuals/m^2^. During the 2021 survey, only two mollusc species (*C. africana* and *T. granifera*) were collected from the two sites on the Olifants River with a total average density of 338 individuals/m^2^. *Tarebia granifera* dominated during both the 2020 and 2021 surveys. During 2021, *T. granifera* contributed to 77.71% and *C. africana* to 22.29% of the mollusc community at OLIF‐1, whilst *T. granifera* contributed to more (91.81%) and *C. africana* to less (8.19%) at OLIF‐2. In 2021, the highest densities of *C. africana* were sampled at OLIF‐1 (193 individuals/m^2^) and OLIF‐3 (183 individuals/m^2^) during 2020, whilst the lowest densities were recorded during 2021 at OLIF‐1 (14 individuals/m^2^) and OLIF‐2 (50 individuals/m^2^). *Physella acuta* was only sampled on the Olifants River during the 2020 survey, with the highest density recorded from OLIF‐3 (five individuals/m^2^) and OLIF‐1 (four individuals/m^2^), whilst OLIF‐2 had a density of two individuals/m^2^, contributing to <1% of the total mollusc communities. No *P. acuta* were found at the two sites on the Olifants River during the 2021 survey.

Apart from two sites (LIMP‐1 and LETA), *T. granifera* was the dominant molluscan species at all sites sampled on the Limpopo River system. Three mollusc species (*C. africana*, *M. tuberculata*, and *T. granifera*) were collected from the two sites on the Limpopo River with a total average density of 283 individuals/m^2^. No molluscs were present at LIMP‐3. *Melanoides tuberculata* was the dominant mollusc at LIMP‐1 (528 individuals/m^2^), followed by *C. africana* (five individuals/m^2^) and *T. granifera* (three individuals/m^2^). At LIMP‐2, *C. africana* and *T. granifera* had similar contributions to the community with higher densities (14 and 15 individuals/m^2^, respectively) than LIMP‐1.

Three species of molluscs (*C. africana*, *P. acuta*, and *T. granifera*) were sampled from the site on the Letaba River (LETA), with a density of 118 individuals/m^2^. The dominant species was *C. africana*, at 61 individuals/m^2^, followed by *T. granifera* at 56 individuals/m^2^ and *P. acuta* with only one individual/m^2^. The entire molluscan community (100%) at LUVU consisted of *T. granifera* (42 individuals/m^2^). This was the only site with a single species and the lowest mollusc diversity (excluding the sites where no molluscs were present). From the remaining three sites on the Limpopo River system, only *C. africana* and *T. granifera* were present. The average mollusc densities at these sites were: 215 individuals/m^2^ (G‐LETA), 137 individuals/m^2^ (MOGA) and 69 individuals/m^2^ (SHIN) with *T. granifera* being the dominant species at all three sites.

Mollusc densities differed significantly (Table 4) between species in each of the three river systems, Phongolo River (*p* = .0097, *χ* ^2^ = 9.37), Olifants River (*p* = .0002, *χ* ^2^ = 10.84) and Limpopo River system (*p* = .0017, *χ* ^2^ = 15.16). This was a result of significantly higher densities of *T. granifera* compared with *M. tuberculata* (*p* = .016) and *P. acuta* (*p* = .049) in the Phongolo River. In the Olifants River, this was a result of significantly higher densities of *T. granifera* compared with *P. acuta* (*p* = .002) densities. From the other sites on the Limpopo River system, these differences again occurred as a result of significantly higher densities of *T. granifera* compared with *M. tuberculata* (*p* = .006) and *P. acuta* (*p* = .001) densities. There were no significant differences found between *T. granifera* and *C. africana* densities. None of the multiple regression analyses run to predict *P. acuta and C. africana* abundances using either the various water quality variables or the *T. granifera* density and size class contributions as predictors were able to significantly predict these species abundances across sites: *P. acuta* versus water quality variables – *F* (5, 10) = .643, *p* = .673, *R* ^2^ = .243; *C. africana* versus water quality variables – *F* (5, 10) = 1.323, *p* = .329, *R* ^2^ = .398; *P. acuta* versus *T. granifera* population variables – *F* (4, 8) = 2.617, *p* = .115, *R* ^2^ = .567; *C. africana* versus *T, granifera* population variables – *F* (4, 8) = 1.777, *p* = .229, *R* ^2^ = .469.

**TABLE 4:** Kruskal–Wallis and Dunn's post hoc test of pairwise differences between the densities of *Tarebia granifera* and other mollusc species sampled on each of the three river systems.

River	Kruskal–Wallis test		*Tarebia granifera* vs.	Dunn's post hoc test
Phongolo River	*p* = .0097*	*χ* ^2^ = 9.37	*Corbicula africana* *Melanoides tuberculate Physella acuta*	*p* = .098 *p* = .016* *p* = .049*
Olifants River	*p* = .0002*	*χ* ^2^ = 10.84	*Corbicula africana* *Physella acuta*	*p* = .458 *p* = .002*
Limpopo River system	*p* = .0017*	*χ* ^2^ = 15.16	*Corbicula africana* *Melanoides tuberculata* *Physella acuta*	*p* = .230 *p* = .006* *p* = .001*

* Indicates significant differences (*p* < .05).

## 4 DISCUSSION

The central aim of the study was to investigate the current distribution, densities, and population size structures of *T. granifera* at selected sites in rivers in north‐eastern South Africa and the potential environmental drivers thereof as well as their potential impacts on native aquatic mollusc diversity in the region. The key findings around the three core questions indicated that *T. granifera* was the dominant species of all molluscs sampled and was also the only species to be present at each of the sites that contained molluscs. Population structures were found to differ between sites and systems with higher newborn recruitment observed during the sampling events in spring. Apart from this observation, size class distributions were independent of specific sites or systems. However, *T. granifera* densities were related to the system sampled. In contradiction with previous reports, no significant relationships were found between any of the *T. granifera* population variables and water quality parameters. The densities reached by *T. granifera* were much higher than those of native as well as other invasive molluscs. Although no direct statistical inference could be made regarding the effects of *T. granifera* population variables on mollusc abundance, the presence of these invasive snails likely had a negative effect on native snail density and diversity.

### 4.1 *Tarebia granifera* density

Invasive species often achieve densities higher than those found in their native areas and potentially have negative impacts on indigenous species (Gallardo et al., 2016; Pearson et al., In press; Prentis et al., 2008). The high densities of *T. granifera* recorded at sites during the present study correspond with previous studies in South Africa that found densities of over 1000 individuals/m^2^ (Appleton et al., 2009; Miranda et al., 2011b). Additionally, the lower densities found during the present study at selected sites in the Limpopo River correspond with the lower densities reported by Makherana et al. (2022) in the Nandoni Reservoir, South Africa. It should be noted that even the low densities recorded during the present study were usually much higher than the densities reported for *T. granifera* (18–193 individuals/m^2^) in its native ranges (Appleton & Nadasan, 2002; Dudgeon, 1980). These lower densities in the Limpopo River may be related to a naturally low carrying capacity in the system at the time of sampling (Makherana et al., 2022). This, however, is unlikely as a previous study found between 27 and 4000 individuals of *T. granifera* were present in the Makuleke Wetlands on the Limpopo River in the northern reaches of the KNP during a study that took place in 2015 and 2016 (de Necker et al., 2023). We hypothesize that this low density is rather related to environmental disturbances, such as drought, flooding, food availability, and increased salinity (Appleton et al., 2009; Miranda et al., 2011b), reducing the population, with the observed lower density, thus an indication of an early invasion period after the disturbance. These findings support our hypothesis that *T. granifera* densities will reflect environmental conditions, with lower densities occurring in less favourable environmental conditions.

One adult *T. granifera* (18–20 mm) can have an average of up to 48.6 unborn juveniles in its brood pouch and larger adults (20–30 mm) have an average of 158 unborn juveniles in their brood pouch. This suggests that only a single snail is needed to start a new population (Miranda et al., 2011b). This provides further insight into how rapidly these invaders can reach high population densities even after population crashes due to environmental disturbances. However, further studies consisting of long‐term and more frequent data collection surveys would be necessary to formally assess and support this observation.

The results from the present study indicated differences in *T. granifera* density between seasons, although these findings should be interpreted with caution as only two sites were sampled over different seasons. Previous studies on the changes in *T. granifera* population densities have been contradictory, with several reporting maximum densities sampled at different times of the year (Appleton et al., 2009; Miranda et al., 2011b). Miranda et al. (2011b) found that *T. granifera* density did not appear to undergo seasonal patterns in an estuarine environment, whereas Makherana et al. (2022) found significant differences in *T. granifera* densities across seasons from a freshwater environment in Nandoni Dam.

Since no significant correlations were found between *T. granifera* density and water quality variables, the decrease in snail density during autumn may likely be attributed to heavy rain/seasonal change and increased water velocity during the wet season (summer). *Tarebia granifera* densities may quickly increase again during the dry season (winter–spring) when nutrients, and thus food availability, increase (Appleton et al., 2009; López‐López et al., 2009). This was also evident in the study by López‐López et al. (2009) that found *T. granifera* populations from the Tuxpam and Tecolutla rivers in the USA had distinct reductions in snail density during the heavy rainy season (summer), where after populations responded quickly during the dry season (winter) when nutrients increased food availability. This may also explain why higher densities of *T. granifera* were present in the Phongolo River during winter–spring (August and September) compared with sites sampled on the Limpopo River system during autumn (April and May), as the Phongolo River catchment was experiencing a significant drought following the severe El Niño event of 2016, resulting in no seasonal flooding during the sampling period (de Necker et al., 2021; de Necker, Brendonck, et al., 2022).

### 4.2 *Tarebia granifera* population size structures

*Tarebia granifera* populations seemed to follow a trend of higher newborn numbers during spring and summer whilst fewer newborn snails were recorded during autumn and winter months. Appleton et al. (2009) found that *T. granifera* populations from KwaZulu‐Natal declined in density by as much as 95% every few years. This decline was attributed to low birth rates during the spring/summer season. However, densities rapidly increased again in only a few months (Appleton et al., 2009). Appleton and Nadasan (2002) also mentioned that there seems to be a peak in *T. granifera* birth during the summer months when it is warmer. *Tarebia granifera* have adapted to minimize mortality of early life stages during adverse events, different sizes of unborn snails have been found in the brood pouches of adult snails, suggesting that these unborn snails may be retained during unfavourable conditions (Miranda et al., 2011b). Larger individuals of *T. granifera* have also been found to have a higher tolerance to desiccation and high salinity during dry events, enabling them to survive until more favourable conditions are reached (Miranda et al., 2011b). Interestingly, Miranda et al. (2011b) reported that some *T. granifera* populations seem to give birth all year round. This suggests that the birth rate may not be seasonal but rather dependent on environmental variables, such as water depth, wet or dry conditions, salinity, temperature, and the availability of food sources (López‐López et al., 2009; Miranda et al., 2011b).

The lower proportion of newborn snails from the Phongolo population might be explained by seasonal environmental changes and overall adverse conditions, as sampling occurred during winter–spring (dry season) at the peak of a strong El Niño event that resulted in a severe drought within the catchment. The study by Miranda et al. (2011b) reported that *T. granifera* populations were able to quickly re‐establish when favourable conditions returned after a period of desiccation, the adult population gave birth to large numbers of newborn snails, and by the end of 2010, the population had recovered in structure and density. Prior research has demonstrated an increased number of unborn snails in the brood pouch during times of environmental stress (increased salinity or desiccation) as well as during times of population recovery when environmental conditions became more favourable (Miranda et al., 2011b). This suggests that *T. granifera* responds to environmental variability by increasing reproductivity and consequently speeding up population recovery (Miranda et al., 2011b). These findings further support our hypothesis that *T. granifera* population size classes reflect environmental conditions as more varied populations were present in more favourable environmental conditions. Additional research using microcosm or mesocosm experiments could provide further clarity on the impact of environmental variables on the population structures of this invasive species.

The average birth rate of 1.89 newborn *T. granifera* per day from the present study corresponds with the reports that adults can give birth to one newborn snail every 12 h (Appleton et al., 2009). This ability to rapidly reproduce and the high environmental tolerance of mature adults play a key role in their success as an invader and ensure the persistence of *T. granifera* within invaded habitats as repeated introductions are not essential for them to successfully colonize new areas (Appleton et al., 2009; Miranda et al., 2011b). From the present study, *T. granifera* samples from only three sites had no newborn snails, although all three of these sites had mature adults. These results are similar to those reported by Miranda et al. (2011a), with higher abundances of smaller size classes (<10 mm) at select sites and during certain times of the year (Austral spring). This is of concern as previous studies have indicated that juvenile snails can have higher impacts on food resources than larger adult snails as juvenile snails consumed more food by mass than adult snails (Miranda et al., 2011a; Tamburi & Martín, 2009).

### 4.3 *Tarebia granifera* population variables versus water quality parameters

Adult *T. granifera* have a higher tolerance for environmental factors, including dry periods, colder temperatures, food availability, and increased salinity, causing lower reproduction in adults during unfavourable conditions (Miranda et al., 2011b). Several studies have reported differences in *T. granifera* densities due to differences in water pH, conductivity, and TDS (total dissolved solids) (e.g. Jones et al., 2017; Makherana et al., 2022). In particular, this species shows a strong positive relationship with conductivity, with higher densities occurring at higher conductivity and TDS (Makherana et al., 2022).

However, this does not seem to be the case in the present study as no significant correlations were found between *T. granifera* density and water quality variables in any of the sampled river systems. Similarly, Miranda et al. (2011b) found that in comparison with other snails, *T. granifera* was the least associated with any of the water quality variables and was the most dominant and widely distributed species from all study areas. We hypothesize that the increased conductivity and TDS may be a result of *T. granifera* ecology and biology, caused by their movement behaviour in and through sediments (Appleton et al., 2009) leading to the resuspension and dissolution of contents such as salts and nutrients (Jolly et al., 2008). This may be particularly true since high densities of benthic gastropods are known to cause sediment and microphytobenthos resuspension (Orvain et al., 2004). However, further comprehensive studies into such effects of *T. granifera* on water quality are necessary to support this observation.

### 4.4 Other mollusc diversity, density and community size structures

*Tarebia granifera* made up 89% of all sampled snails and was the only snail species present at each of the 14 sites that had live molluscs present. These findings are similar to those of previous studies where *T. granifera* have been reported to often be the dominant invertebrate species within invaded aquatic communities (Appleton et al., 2009; de Necker et al., 2021, 2023; Majdi et al., 2022). As a result, *Tarebia granifera* is likely to have a negative impact on the community structures of native snail species, resulting in a loss of biodiversity and declines in the abundance of native species (Appleton et al., 2009; Jones et al., 2017; Miranda et al., 2011a).

Importantly, *T. granifera* has been reported to cause declines and extinctions in native snail populations from Cuba, Venezuela, and Puerto Rico (Pearson et al., In press) as well as elsewhere in South Africa (Appleton et al., 2009; Jones et al., 2017; Miranda et al., 2011a, 2011b; Pearson et al., In press). The present study found evidence of three native snail species that seem to have disappeared from their habitat and supports our hypothesis that higher densities of *T. granifera* will exhibit a negative impact on benthic mollusc diversity. Empty shells of Planorbidae were sampled from the Phongolo River, *Bulinus tropicus* (Krauss, 1848) from the Shingwedzi River site and *B. forskalii* shells were collected from the MOGA site. The native *M. tuberculata* was also absent from several sites during the present study. In addition, historical records (FBIS, 2020; Oberholzer & Van Eeden, 1967; Pretorius et al., 1975; Wolmarans & de Kock, 2006) of snail species from the sites sampled during the present study indicated that several mollusc species (see Appendix 1) including *Biomphalaria* and *Bulinus* species were absent from the Limpopo, Luvuvhu, Olifants, Letaba and Phongolo River sites. Similarly, Miranda et al. (2011b) found that historically recorded snail species (*M. tuberculata* and *Bellamya capillata* Frauenfeld, 1865) were absent from Lake Sibaya during their study, and attributed this to the high densities of the invasive *T. granifera*. Interestingly, the native *C. africana* coexisted with *T. granifera* at all sites sampled during the present study, coexisting even at sites where *T. granifera* had the highest recorded densities. López‐López et al. (2009) reported similar findings from the Tuxpam and Tecolutla rivers in the USA, where *T. granifera* and a *Corbicula* sp. (both invasive in the USA) coexisted and dominated in invaded habitats whilst several native species were missing or had limited distribution with low densities. These findings may be explained by differences in the habitat distribution, feeding strategies, and diet preferences of these two molluscan species (López‐López et al., 2009), thus preventing direct competition between them.

The absence of historically recorded native species, and their overall low diversity from the river systems sampled during the present study is of concern and is quite likely linked to the ability of *T. granifera* to reach high densities and drive away other species (Appleton et al., 2009; Makherana et al., 2022). However, this requires further investigation before a definitive conclusion may be reached (Pearson et al., In press). We recommend that in vivo studies (microcosms or mesocosm) be performed whereby the direct impacts of *T. granifera* are measured regarding other snails of interest.

Our study findings supported two of our hypotheses as we found higher densities and more varied population structures of *T. granifera* in more favourable environmental conditions. This invasive species has the ability to increase reproduction, and consequently, population recovery, when conditions become favourable, contributing to their invasion success. The *T. granifera* population structures also reflected an established population, with individuals across all the size classes at sampled sites. However, contrary to previous studies and our hypothesis, we found *T. granifera* densities were not driven by increased conductivity and TDS. Our results instead indicated that the increase in the conductivity and TDS could potentially be attributed to the benthic biology, and resultant bioturbation, of *T. granifera*. This study further found evidence of the disappearance of native aquatic snail species from the sampled ecosystems that may well be linked to the presence as well as high densities of *T. granifera*. At the high densities demonstrated in this study, the feeding of *T. granifera* may have further ecosystem‐wide implications on the food web dynamics of invaded ecosystems, and further investigation is needed to determine how *T. granifera* affects native aquatic biota.

Sampling limitations, including seasonality, location (study area), and a lack of regular sampling events over a longer period, thus limit our ability to draw concrete conclusions regarding the population dynamics of *T. granifera* or the environmental drivers behind them. However, this is the first study to determine the current distribution of *T. granifera* throughout the Limpopo River catchment in South Africa and the first to characterize and provide insights into the densities and population structures of these invasive snails within rivers in north‐eastern South Africa.

Future studies should investigate environmental drivers in order to gain more information on how *T. granifera* populations react to environmental changes and determine the environmental factors that influence their densities as well as their population size structures. It is also recommended that long‐term, regular sampling campaigns over different seasons are conducted to gain insight into the ecological drivers that influence the densities and population size structures of *T. granifera* within invaded ecosystems. A better understanding of how their populations will react towards environmental changes can lead to the development, implementation, and management of control strategies. Additional investigations are necessary to understand how *T. granifera* interacts with native snails as well as other benthic communities such as diatoms and macroinvertebrates. This would provide insights into the role of this invader in causing local extinctions and declines in native snail species abundances in invaded ecosystems.

## AUTHOR CONTRIBUTIONS

**Ruan Gerber:** Conceptualization (equal); methodology (equal); supervision (equal); writing – review and editing (equal). **Johannes J. Pearson:** Formal analysis (equal); investigation (equal); methodology (equal); visualization (equal); writing – original draft (equal). **Victor Wepener:** Conceptualization (equal); funding acquisition (equal); project administration (equal); resources (equal). **Wynand Malherbe:** Conceptualization (equal); investigation (equal); resources (equal); supervision (equal); writing – review and editing (equal). **Lizaan de Necker:** Conceptualization (equal); formal analysis (equal); funding acquisition (equal); supervision (equal); visualization (equal); writing – review and editing (equal).

## CONFLICT OF INTEREST STATEMENT

The authors have no conflict of interest to declare.

## Data Availability

All data are available via the Dryad platform. Gerber, Ruan et al. (Forthcoming 2024). Distribution, population dynamics and potential impacts of the invasive snail, *Tarebia granifera* in aquatic ecosystems of north‐eastern South Africa [Dataset]. Dryad. https://doi.org/10.5061/dryad.w0vt4b90c.

## APPENDIX 1

### List of aquatic snail species historically recorded from the river systems sampled during the present study

**Table:**

River	Aquatic snails	Date	Reference
Phongolo	*Afrogyrorbis natalensis* (Krauss, 1848)	1971	Pretorius et al. (1975)
	*Chambardia petersi* (von Martens, 1860)	1971	Pretorius et al. (1975)
	*Chambardia wahlbergi* (Krauss, 1848)	1971	Pretorius et al. (1975)
	*Bellamya capillata* (Frauenfeld, 1865)	1971	Pretorius et al. (1975)
	*Biomphalaria pfeifferi* (Krauss, 1848)	1971	Pretorius et al. (1975)
	*Bulinus africanus* (Krauss, 1848)	1971	Pretorius et al. (1975)
	*Bulinus forskalii* (Ehrenberg, 1831)	1971	Pretorius et al. (1975)
	*Bulinus natalensis* (Küster, 1841)	1971	Pretorius et al. (1975)
	*Cleopatra ferruginea* (Lea & Lea, 1851)	1971	Pretorius et al. (1975)
	*Corbicula africana* (Krauss, 1848)	1971	Pretorius et al. (1975)
	*Eupera ferruginea* (Krauss 1848)	1971	Pretorius et al. (1975)
	*Pettancylus cawstoni* (Walker, 1903)	1971	Pretorius et al. (1975)
	*Gyraulus costulatus* (Krauss, 1848)	1971	Pretorius et al. (1975)
	*Lanistes ovum* (Peters, 1845)	1971	Pretorius et al. (1975)
	*Pseudosuccinea columella* (Say, 1817)	1971	Pretorius et al. (1975)
	*Radix natalensis* (Krauss, 1848)	1966	Oberholzer and Van Eeden (1967)
	*Melanoides tuberculata* (Müller, 1774)	1966	Oberholzer and Van Eeden (1967)
	*Afropisidium pirothi* (Jickeli, 1881)	1971	Pretorius et al. (1975)
	*Segmentorbis kanisaensis* (Preston, 1914)	1971	Pretorius et al. (1975)
	*Coelatura mossambicensis* (Martens, 1860)	1971	Pretorius et al. (1975)
Crocodile	Burnupidae (Rafinesque, 1815)	2016	FBIS (2020)
	*Afrogyrorbis* (Studer, 1820)	1957	FBIS (2020)
	*Bulinus* (Müller, 1781)	2016	FBIS (2020)
	*Burnupia* (Walker, 1912)	1964	FBIS (2020)
	*Unio caffer* (Krauss, 1848)	—	FBIS (2020)
	*Corbicula africana* (Müller, 1774)	2015	FBIS (2020)
	*Pettancylus* (Walker, 1903)	1964	FBIS (2020)
	*Gyraulus* (Charpentier, 1837)	1964	FBIS (2020)
	*Radix natalensis* (Krauss, 1848)	1957	FBIS (2020)
	*Physella acuta* (Draparnaud, 1805)	2016	FBIS (2020)
	*Planorbis* (Müller, 1773)	1965	FBIS (2020)
	Sphaeriidae (Deshayes, 1855)	2016	FBIS (2020)
	*Sphaerium capense* (Krauss, 1848)	2016	FBIS (2020)
	*Tarebia granifera* (Lamarck, 1816)	2017	FBIS (2020)
	*Unio caffer* (Krauss, 1848)	1966	Oberholzer and Van Eeden (1967)
Limpopo	*Biomphalaria pfeifferi* (Krauss, 1848)	1962	FBIS (2020)
	*Bulinus* (Müller, 1781)	1962	FBIS (2020)
	*Corbicula* (Megerle von Mühlfeld, 1811)	1962	FBIS (2020)
	*Pettancylus* (Walker, 1903)	1962	FBIS (2020)
	*Gyraulus* (Charpentier, 1837)	1962	FBIS (2020)
	*Lymnaea* (Lamarck, 1799)	1962	FBIS (2020)
	*Melanoides tuberculata* (Müller, 1774)	1962	FBIS (2020)
	*Euglesa* (Pfeiffer, 1821)	1962	FBIS (2020)
	*Antigona lamellaris* (Schumacher, 1817)	—	FBIS (2020)
Matlabas			No data found
Lephalala	*Biomphalaria* (Preston, 1910)	1960	FBIS (2020)
Mogalakwena	*Biomphalaria pfeifferi* (Krauss, 1848)	1960	FBIS (2020)
	*Bulinus* (Müller, 1781)	1960	FBIS (2020)
Sand	*Lymnaea* (Lamarck, 1799)	2015	FBIS (2020)
	Sphaeriidae (Deshayes, 1855)	2016	FBIS (2020)
	*Bulinus* (Müller, 1781)	2016	FBIS (2020)
	*Corbicula* (Megerle von Mühlfeld, 1811)	2016	FBIS (2020)
	*Physella acuta* (Draparnaud, 1805)	2016	FBIS (2020)
	*Tarebia granifera* (Lamarck, 1816)	2017	FBIS (2020)
Luvuvhu	*Bulinus* (Müller, 1781)	2016	FBIS (2020)
	*Burnupia* (Walker, 1912)	2017	FBIS (2020)
	*Afrogyrorbis natalensis* (Krauss, 1848)	2015	FBIS (2020)
	*Corbicula* (Megerle von Mühlfeld, 1811)	2017	FBIS (2020)
	*Lymnaea* (Lamarck, 1799)	2016	FBIS (2020)
	*Physella acuta* (Draparnaud, 1805)	2016	FBIS (2020)
	Planorbidae (Rafinesque, 1815)	2016	FBIS (2020)
	Sphaeriidae (Deshayes, 1855)	2017	FBIS (2020)
	*Sphaerium capense* (Krauss, 1848)	2016	FBIS (2020)
	*Tarebia granifera* (Lamarck, 1816)	2017	FBIS (2020)
*Biomphalaria* (Preston, 1910)	Olifants	1965	FBIS (2020)
	*Bulinus* (Müller, 1781)	2015	FBIS (2020)
	*Burnupia* (Walker, 1912)	1965	FBIS (2020)
	*Corbicula africana* (Krauss, 1848)	1959	FBIS (2020)
	*Corbicula africana* (Krauss, 1848)	2017	FBIS (2020)
	*Eupera* (Bourguignat, 1854)	1986	FBIS (2020)
	*Pettancylus* (Walker, 1903)	1965	FBIS (2020)
	*Gyraulus costulatus* (Krauss, 1848)	1960	FBIS (2020)
	*Lymnaea* (Lamarck, 1799)	1965	FBIS (2020)
	*Melanoides tuberculata* (Müller, 1774)	2015	FBIS (2020)
	*Physella acuta* (Draparnaud, 1805)	2015	FBIS (2020)
	*Euglesa* (Pfeiffer, 1821)	2016	FBIS (2020)
	Planorbidae (Rafinesque, 1815)	2017	FBIS (2020)
	Sphaeriidae (Deshayes, 1855)	2016	FBIS (2020)
	*Tarebia granifera* (Lamarck, 1816)	2017	FBIS (2020)
	*Unio caffer* (Krauss, 1848)	1966	Oberholzer and Van Eeden (1967)
Great Letaba	*Eupera ferruginea* (Krauss, 1848)	1966	Oberholzer and Van Eeden (1967)
Letaba	*Aplexa marmorata* (Guilding, 1828)	2006	Wolmarans and de Kock (2006)
	Ampullariidae (Grey, 1824)	2017	FBIS (2020)
	Burnupidae (Walker, 1923)	2016	FBIS (2020)
	*Chambardia wahlbergi* (Krauss, 1848)	1932	FBIS (2020)
	*Biomphalaria pfeifferi* (Krauss, 1848)	2017	FBIS (2020)
	*Bulinus* (Müller, 1781)	2016	FBIS (2020)
	*Bulinus forskalii* (Ehrenberg, 1831)	2001	Wolmarans and de Kock (2006)
	*Burnupia* (Walker, 1912)	2016	FBIS (2020)
	*Corbicula africana* (Krauss, 1848)	2017	FBIS (2020)
	*Corbicula natalensis* (Cessin, 1879)	1935	FBIS (2020)
	Etheriidae (Deshayes, 1830)	2016	FBIS (2020)
	*Lanistes ovum* (Peters, 1845)	1935	Wolmarans and de Kock (2006)
	*Radix natalensis* (Krauss, 1848)	2015	FBIS (2020)
	*Pseudosuccinea columella* (Say, 1817)	2006	Wolmarans and de Kock (2006)
	*Melanoides tuberculata* (Müller, 1774)	2006	Wolmarans and de Kock (2006)
	*Melanoides victoriae* (Dohrn, 1865)	2006	Wolmarans and de Kock (2006)
	Neritidae (Rafinesque, 1815)	2016	FBIS (2020)
	*Physella acuta* (Draparnaud, 1805)	2017	FBIS (2020)
	*Euglesa* (Pfeiffer, 1821)	2016	FBIS (2020)
	Planorbidae (Rafinesque, 1815)	2015	FBIS (2020)
	Sphaeriidae (Deshayes, 1855)	2016	FBIS (2020)
	*Tarebia granifera* (Lamarck, 1816)	2017	FBIS (2020)
	*Unio caffer* (Krauss, 1848)	2016	FBIS (2020)
Shingwedzi	*Lanistes ovum* (Peters, 1845)	1966	Oberholzer and Van Eeden (1967)
